# Understanding the complex relationships among actors involved in the implementation of public-private mix (PPM) for TB control in India, using social theory

**DOI:** 10.1186/s12939-018-0785-1

**Published:** 2018-06-07

**Authors:** Solomon Salve, Kristine Harris, Kabir Sheikh, John D. H. Porter

**Affiliations:** 1 Health Governance Hub Public Health Foundation of India, Plot No. 47, Sector 44, Institutional Area, Gurgaon, Haryana 122002 India; 2 Health Governance Hub, Public Health Foundation of India, Plot No. 47, Sector 44, Institutional Area, Gurgaon, Haryana 122002 India; 3 Department of Global Health and Development, London School of Hygiene and Tropical Medicine, Keppel Street, London, WC1E 7H UK; 4 Departments of Clinical Research and Global Health and Development, London School of Hygiene and Tropical Medicine, Keppel Street, London, WC1E 7H UK

**Keywords:** Public-private mix, TB programme, Public private partnership, Social theory, Anthropology

## Abstract

**Background:**

Public Private Partnerships (PPP) are increasingly utilized as a public health strategy for strengthening health systems and have become a core component for the delivery of TB control services in India, as promoted through national policy. However, partnerships are complex systems that rely on relationships between a myriad of different actors with divergent agendas and backgrounds. Relationship is a crucial element of governance, and relationship building an important aspect of partnerships. To understand PPPs a multi-disciplinary perspective that draws on insights from social theory is needed. This paper demonstrates how social theory can aid the understanding of the complex relationships of actors involved in implementation of Public-Private Mix (PPM)-TB policy in India.

**Methods:**

Ethnographic research was conducted within a district in a Southern state of India over a 14 month period, combining participant observations, informal interactions and in-depth interviews with a wide range of respondents across public, private and non-government organisation (NGO) sectors.

**Results:**

Drawing on the theoretical insights from Bourdieu’s “theory of practice” this study explores the relationships between the different actors. The study found that programme managers, frontline TB workers, NGOs, and private practitioners all had a crucial role to play in TB partnerships. They were widely regarded as valued contributors with distinct social skills and capabilities within their organizations and professions. However, their potential contributions towards programme implementation tended to be unrecognized both at the top and bottom of the policy implementation chain. These actors constantly struggled for recognition and used different mechanisms to position themselves alongside other actors within the programme that further complicated the relationships between different actors.

**Conclusion:**

This paper demonstrates that applying social theory can enable a better understanding of the complex relationship across public, private and NGO sectors. A closer understanding of these processes is a prerequisite for bridging the gap between field-level practices and central policy intentions, facilitating a move towards more effective partnership strategies for strengthening local health systems. The study contributes to our understanding of implementation of PPP for TB control and builds knowledge to help policy makers and programme managers strengthen and effectively implement strategies to enable stronger governance of these partnerships.

## Background

Public-Private Partnerships (PPP) are increasingly promoted in India and globally as an innovative strategy for strengthening local health systems [1]. Such partnerships are specifically recognized to be important in the significant scaling up of essential services needed to achieve the national and the global visions espoused by Universal Health Coverage (UHC), itself highlighted within the Sustainable Development Goals (SDGs) [2]. For the purpose of this paper, partnership is broadly considered to indicate inter-organizational relationships and collaborations involving individuals and organizations from the public, private and NGO sectors for delivering better healthcare. Engaging all relevant healthcare providers in TB care and control through public-private mix (PPM)^1^ approaches is, for example, considered an essential component of the WHO’s Stop TB Strategy [3, 4]. As per the Global TB report 2017 India is one among the five countries (in descending order - India, Indonesia, China, the Philippines and Pakistan) having the largest number of incident cases in 2016 which together accounted for 56% of the global total [5]. In 2016, the estimated incidence of TB in India was approximately 2.8 million accounting for about a quarter of the global burden of TB [6]. The Revised National Tuberculosis Control Programme (RNTCP) in India employs a comprehensive approach, involving a wide range of public and private providers previously not linked to the TB control programme [7, 8]. National PPM-TB policy for engagement with the private sector has been a core component in the TB control strategy for more than a decade [9, 10]. The partnership initiatives under the PPM-TB policy broadly aims to strengthen the referral systems between the public, the private and NGO sectors to reach out to more patients and to provide standardized diagnosis and treatment [11]. RNTCP has established Designated Microscopy Centres (DMCs) across the country, but accessibility to DMCs in difficult to reach areas remains to be sub-optimal. It is envisaged that NGO/private provider supported sputum collection centres can be established to enhance equity and provide ease of accessibility to patients [12].

In order to maximize the health benefits of partnerships and strengthen local health systems, Widdus (2003) asserts that it is important to understand the implementation of public-private partnerships [13]. This can provide the basis for thinking through how to enable stronger governance of these partnerships - understanding governance as encompassing concern for the management of these relationships in implementation to support the achievement of their goals [14]. However, current evaluations of PPPs mostly focus on global and national level experience [15, 16], with limited attention paid to how ‘individual partnerships’ work and play out at the local level – that is, considering how they are perceived and how they are put into practice [17, 18]. Available studies of PPP implementation [19–22] also fall short in providing an adequate picture of the day to day implementation of partnerships at the local level, demonstrating little understanding of the relationships between different actors. Yet partnerships are complex systems that rely on relationships between a myriad of different actors with divergent agendas and backgrounds. In their analysis of Indian TB partnerships, Ogden and Porter (2000), for example, placed emphasis on relationship building as an important aspect of partnerships [23]. Similarly, Ramiah and Reich (2006) in their study of HIV/AIDS partnership in Botswana, identified the need for partners to build relationships, not only at a technical level but also at operational levels [24]. More generally, relationships between actors are not technically determined [25], but are rather constantly influenced by the relational elements that guide human behaviour and social relationships [26–28]. In order to understand the implementation of PPPs, it is important, then, to understand the factors influencing relationships between the actors involved within them. The complexity of these relationships can best be understood by applying multi-disciplinary perspectives drawing on insights from social theory. Although several studies have demonstrated the relevance of social theory, and anthropological perspectives in understanding health policy-making and implementation [25, 29–37], the use of social theory remains limited in policy implementation studies.

Against this backdrop, this paper aims, first, to demonstrate how social theory can aid understanding of the complex relationships of actors involved in the implementation of public-private partnerships – focusing on the implementation of the national policy for engaging the private sector in Tuberculosis control in India. Relationship is a crucial element of governance, and relationship building an important aspect of partnerships. So the paper also draws conclusions from this analysis relevant to the governance of PPPs.

## Methods

### Theoretical framing

To aid inquiry into the local level implementation of PPPs, this paper draws on theoretical insights from Pierre Bourdieu’s ‘theory of practice’. Bourdieu (a French anthropologist and social theorist) was interested in understanding the complex relationships between the individual and society, considering the interaction between agency (individuals’ independent ability to make decisions and free choices [38]) and structure (‘structure’ is responsible for shaping individual behavior, and that it restricts the choices made by individuals [39]). In the process of his studies, he conceptualized four interrelated concepts of *field*, *habitus, capital* and *doxa* to explain how social practices are constructed in and through the relationships within particular areas of social life [40]. Pierre Bourdieu’s work focuses on the relation between structure and agency [41–43]. He was in favour of finding a balance between the two positions, and viewed the relationship between structure and agency as dynamic and recursive – structure influences human behaviour, and humans are capable of changing the social structures they inhabit.

According to Bourdieu, individuals (actors) have positions in groups, groups have positions in field and fields are positioned in relation to each other in society. Fields can be defined in different ways – e.g., a geographic location, set of activities, organization, religion, or lifestyle, etc. However, a field is essentially a specific space of relationships, a ‘site of struggle’, a type of battlefield where the positions in it compete over, contest, construct, influence, and have power to improve their positions [40]. His notion of ‘habitus’ refers to ‘cognitive structures’ that individuals use to deal with the outer social world and these structures are formed based on their past experiences within and across social fields [42]. Another important concept is that of ‘capital’ [44], referring to ‘the resources and assets within particular fields which actors struggle to acquire and through which they “play the game” within each field’ [40]. Bourdieu extends his concept of capital beyond the notion of material assets to: economic capital (money and property); cultural capital (cultural goods and services, including educational credentials); social capital (networks and acquaintances); and symbolic capital (honor, prestige or recognition) [45]. Bourdieu’s concept of capital is useful for this analysis of PPP implementation because he explicitly links social capital to ‘interconnectedness’ – to the networks and relationships a person is part of and the position they hold within such networks [44]. The last concept of ‘doxa’ is the combination of unstated, unconscious, taken for granted assumptions, norms, and beliefs that are perceived by the individual as ‘common sense’ or self-evident [42]. In this paper, we specifically apply Bourdieu’s theoretical concepts of *field and capital* to gain a deeper understanding of the social processes involved in the implementation of PPM-TB policy.

### Study setting

The study was conducted within a district in a Southern state of India due to its decade old history of PPM for TB control. To safeguard the anonymity and confidentiality of the respondents the study site will be left unnamed. More specifically, in order to conduct thorough in-depth and focused work, fieldwork was conducted in one of the nine TB Units (TU) established within the study district.

Each TU covers an approximate population of 0.5 million and each consists of four to five Designated Microscopy Centres (DMCs)^.^^2^. In consultation with the District programme managers, the DBR TU was selected as an area for detailed exploration of partnership implementation. The DBR TU covers a population of 405,230 (0.4 million) and is spread across four DMCs, one of them being run by NGO-A in partnership with the TB programme. In addition, there were other government run health facilities^3^ and a huge numbers of individual private clinics (approx. 300), and private diagnostic centres operating in the nearby areas surrounding the DMCs. There were also substantial numbers of private nursing homes, and a couple of big corporate hospitals.

### Data collection

Fieldwork is at the heart of the discipline of anthropology, wherein an anthropologist goes to the field and stays with the people whom s/he wants to study [46]. As Hammersley [47] states, ‘The task [of ethnographers] is to document the culture, the perspectives and practices, of the people in these settings. The aim is to “get inside” the way each group of people see the world’ (quoted in Reeves et al., 2008:337). The fieldwork period for this study encompassed making links and interacting with the respondents across public, private and non-government organisation (NGO) sectors, engaging with them on a day to day basis, and becoming part of their lives. The respondents included: frontline staff from the TB programme; District Programme Managers; Private Practitioners (General Physicians and Chest physicians) and representatives of Medical Associations; and Partner NGOs.

The research was conducted over a period of fourteen months from October 2010 up until December 2011, collecting information at the field site using various qualitative methods, including: participant observations, informal interactions, in-depth Interviews, and focus group discussions (FGDs). Interview guides focused on understanding of partnerships from the respondents’ perspective and how they experienced its implementation at district level. Participant observation served as an important technique for cross-checking and verifying the information provided by participants during informal interactions or individual interviews. Meticulous notes were maintained in field diaries to capture all information that was heard and observed, and to build reflective thoughts upon them. Taking field notes of all that was seen and heard was intertwined with the entire process of data collection, and proved essential to bring out the rich context of the data collected [48]. A total of 68 in-depth Interviews were conducted in English and Hindi language, were tape recorded, and preceded by informed verbal/written consent.

### Data analysis

All recorded interviews were transcribed word for word. In analyzing the policy at the ‘implementation’ level, we relied on thematic analysis [49]. Data coding was done using thematic analysis and then constantly compared with data from participant observation and notes from field diaries. Themes were identified using a manual method by back and forth reading of data, without using any computer program. Commonalties, meanings and patterns within the interview transcripts and field were identified. These commonalities were then coded, paragraph by paragraph.

Given the exploratory nature of the research, we opted to use an inductive and interpretive approach in analysis to generate, first, what Clifford Geertz calls ‘thick description’ [50] of day to day work patterns and behaviour of frontline actors; their working relations and their experience of the entire partnership process. However, the description itself does not provide an explanation or interpretation of observed actions and behaviour. To take us from ‘thick descriptions’ to the development of empirically-based, real life representative and insightful explanations, we used Bourdieu’s theoretical concepts of *field and capital* to interpret our findings. The application of social theories in this way ‘provide complex and comprehensive conceptual understandings of things that cannot be pinned down: how societies work, how organisations operate, why people interact in certain ways’ [51].

### Ethical considerations

Close attention was paid in respecting ethical considerations for social science research [52]. In a formal interview setting the right to informed consent was strictly observed: written or verbal consent was sought from the respondents giving them as much information about the purpose of the interview, the estimated time required, and the outcome of the study. Measures to safeguard the anonymity and confidentiality of the respondents were observed at all times during the fieldwork and data analysis process. Ethical clearance for this study was obtained from the Institutional Ethics Committee of the London School of Hygiene and Tropical Medicine (LSHTM), London, UK and the Local Indian Ethics Committee.

## Results and discussion

**Fig. 1:**
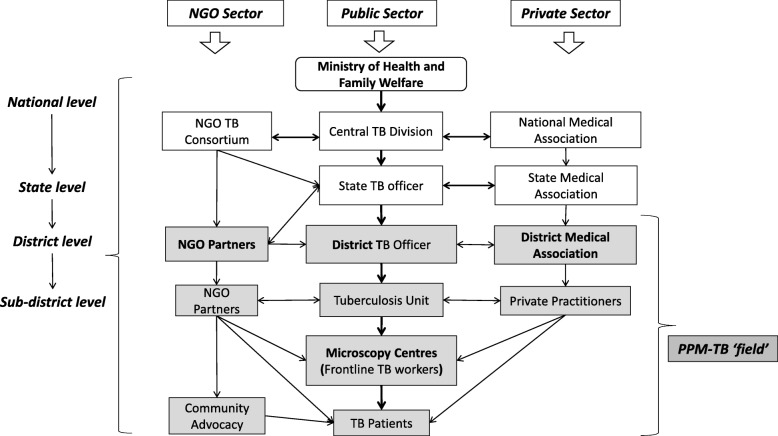
PPM-TB Implementation at the Study Site

The result section has been divided into four major themes in line with the theoretical insights from Bourdieu’s ‘theory of practice’. The RNTCP in India is one of the 13 National Health Programmes implemented by the Department of Health, under the Ministry of Health and Family Welfare (MOHFW). The RNTCP is led by the Central TB Division (CTD) that coordinates the implementation of the programme (Fig. 1). The state, district and sub-district, and peripheral health institutions then implement the TB programme. At the district and sub-district levels, the RNTCP is well integrated into the general health system and can be considered the crucial level of health service delivery, where vertical health programmes and policy intentions are translated into practices [53]. NGOs can collaborate with a programme and adopt schemes as large as running Culture and DST labs, to schemes as small as running a sputum collection centre. Similarly, private practitioners (PPs) can refer suspected TB cases for sputum samples to RNTCP designated microscopy centre (DMC) and, if willing, can act as Directly Observed Treatment Short-course (DOTS) providers for patients diagnosed with TB and initiated on DOTS.

### Conceptualising PPM-TB as a ‘field’

For Bourdieu, it is essential to study the social space (field) where interactions, transactions, and events take place. Drawing on Bourdieu’s concept of field, here we will consider PPM-TB as ‘the field’ itself, where various individuals come to play a role in relation to each other by taking certain positions. The PPM-TB policy guidelines encourage the public sector (RNTCP) to strengthen the referral systems (Fig. 1) between the public, private and NGO sectors, to provide standardised diagnosis and treatment service, and improve the quality of frontline service delivery [54]. Over the years, the PPM-TB policy has evolved and its scope has broadened to include actors across different sectors and at different levels having varying roles and responsibilities (Table 1).

**Table 1:** Role & Responsibility of Actors within the PPM-TB Policy

Actors	Role & Responsibility
District TB Officers (DTOs)	• Key person in programme implementation, holding crucial responsibility for: identifying the partners; developing links with them; and maintaining sustainable partnerships. • He or she also holds the responsibility of calling the District TB Control Society meetings on a quarterly basis and for inviting the partner NGOs to these meetings.
Frontline TB Workers (FTWs)	• Provide feedback to PPs referring TB suspects ○ If the patient doesn’t have TB, then the FTW gives that feedback to the referring PP; ○ If the patient is diagnosed with TB, the FTW then requests the referring PP to be a DOTS provider. ○ If the PP shows unwillingness, then in such cases the FTWs take consent from the referring PPs and place the patient’s box in another nearby DOTS centre. The FTW then counsels the patient that if he/she has any other problems, s/he should see the referring PP for any other side drugs other than TB medicines. • Identify a DOTS provider depending on the patient’s willingness and convenience ○ A DOTS provider could be anyone, from a shop keeper to a qualified medical practitioner. ○ Once the patient shows stability in treatment, and if willing, then the field staff shifts his/her treatment box to a DOTS centre nearest to his/her house. ○ They further explain the DOTS provider that the patient’s need to be given drugs on alternate days. • Follow up with DOTS providers ○ The FTW visits regularly to follow up on the case until treatment is completed. ○ The FTW then reports the successful completion of the treatment to the STS. • Provide honorarium to DOTS providers ○ The FTW updates the STS about the DOTS providers’ supervision. ○ The STS prepares a list of all such PPs every quarter and sends it to the District TB office for release of the PP honorarium. ○ Within 3 to 6 months, the FTW receives a Cheque in hand, which he hands over to the DOTS providers in person and take their signatures on the vouchers to be returned back to the District TB office as a sign of proof.
Private Practitioners (PPs)	• The PP can get involved in a single activity or in multiple activities depending on his or her capacity, interest and the requirements of the programme. ○ *Be a DOTS provider* - the PPs are expected to ensure follow-up sputum collection and late patient retrieval, as well as to maintain RNTCP records for the patients and to permit on-site monitoring by RNTCP supervisory staff as per RNTCP guidelines. ○ *Be a referring provider*- refer TB suspects for diagnosis and treatment, irrespective of whether the client is diagnosed as having TB in private labs or not. ○ Be a DOTS provider, and refer TB suspects. • Practitioners who support as DOTS providers receives an honorarium of Rs. 250 per case (US$ 3.9) that has been successfully completed, whereas, in the case of MDR, the amount goes up to Rs. 1000 per case (US$ 15.9) [54].
Non-Governmental Organisations (NGOs)	• NGOs play an important role in supporting the RNTCP by making treatment more accessible to TB patients through various strategies and programmes, including community-based DOTS. • The RNTCP maintains a formal relationship with NGOs through signing a memorandum of understanding (MoU). • As per the revised schemes for PP/NGO involvement, NGOs can opt for a single scheme or multiple schemes depending upon their experience and capacity. ○ The RNTCP pays on a quarterly-basis for the particular scheme that is opted for. ○ The duration of agreement is one year and is renewable. ○ The NGOs need to re-apply to the District TB Office for funding each year. • The applications are made to the district TB offices, and are renewed only after being signed by the District Magistrate/Collector.

The policy guidelines for the involvement of PPs and NGOs, highlight the crucial role of the District TB Officer (DTO) and the Frontline TB Workers (FTWs) in building and maintaining sustainable partnerships. In addition to district programme managers, FTWs working in the TB units and designated microscopy centres (DMCs), are also seen as the ‘face’ of the programme at the field level. They come into day-to-day contact with the PPs and NGOs to develop both formal and informal partnerships. Although the FTWs role in PP involvement may appear to be quite mechanical, it nevertheless requires a lot of time and effort to build relationships with the PPs and then subsequently to maintain those relationships.

Although we might conceive of the PPM-TB as ‘the field’, it also represents an intersection between the relatively discrete fields of the public, private and NGO sectors. These three sectors tend to be considered as fields, however, within these fields there are several smaller fields that can’t be ignored. For example, within the public sector field, a TB unit could be seen as a field in itself or even district level TB activity could be perceived as a field. Similarly, in the private sector, the Indian Medical Association (IMA) could be seen as a field.

### Individuals and ‘capital’ resources

Individuals operating across fields had varying expertise and, drawing on Bourdieu’s thinking, possessed different types of capital resources.

#### ‘We are the pillars of the programme…’

The FTWs were the ones who knew the ins and outs of the programme at the implementation stage. They identified themselves as ‘pillars of the programme’ being part of the wider community whom they were serving, and further considered their responsibility not only towards the patients alone, but also towards society itself. The TB programme was not merely viewed as a workplace, but also as an opportunity to serve the community, to learn new things that would be treasured for a lifetime. A senior FTW, who had been a regular government employee and was now part of the TB programme, expressed his feelings of working with the TB programme:

…but in this entire service, the TB service will remain as a memory. Apart from all government employees in all other programmes, I understand that the one who is working in TB is the one working hard and no one else works like him. Work happens promptly in the TB programmes. [Interview, Multi-Purpose Health Supervisor-3]

FTWs asserted that TB being a very serious problem, those working in the TB programme needed to have a service-oriented approach. They regarded their intentions to be service-oriented describing it as *‘manava seva madhava seva’* [‘Service to man is service to God’] and in an FGD collectively claimed to be working with ‘full loyalty’. All FTWs felt ‘satisfied’ that their hard work not only healed the patients’ illnesses but also brought happiness into their patients’ lives. They saw the reward for their hard work in terms of the long-term relationships that they developed with the patients.

*…*Above all, our patients get cured. They give us a blessing that is good for us. What is money? Even if the patient says, *"Namaste Anna"* ['hello brother'], that is more satisfying… [Interview, TB Health Visitor-1]

Being pillars of the programme, they were in close relationship with the community and with patients and regarded themselves as the crucial staff in the delivery of the programme. Similarly, NGOs who were working in close proximity with the community and patients, identified themselves as the vital link in the TB programme.

#### ‘We have a watchdog role …’

NGOs perceived themselves as ‘true partners’ to the programme. They took pride in their contribution in bringing a high level of TB awareness among the community; for establishing linkages between community and the government; for developing the community DOTS programme; and for advocating changes in the RNTCP policy. In terms of the policy continuum, NGOs saw themselves positioned ‘at the service end’ that enabled them to work closely with the ‘common man’. The phrase of reaching the “common man” also reflected their intention of reaching those marginalised, vulnerable and beyond the reach of public sector..

…We have administrative machinery from top to bottom to deliver the services to the common man. Unfortunately, they don’t reach the right people who should benefit from such services. So we have a watchdog role, to see that these services reach the right man... [Interview, Chief Executive Officer, NGO-C)

Partnering NGOs, however, saw their role not only in contributing at the level of service provision but also at the level of policy revision. Over a decade ago, NGO-A had initiated a pilot experiment establishing a sputum collection centre (SCC) in a remote and difficult to reach area. This initiative came under one of their projects funded by an international funder. After evaluating their initiatives, they found that the concept of the SCC was practical and feasible, and later advocated for SCC at state and national level in several discussion forums. They were finally able to contribute this concept of an SCC as a policy component within the TB programme.

...We have got some successful models. One, the sputum collection centre, which we started in early 2000, has now been included into the revised NGO/PP policy guidelines... [Interview, Chief Executive Office, NGO-A]

Reflecting on the above two sub-themes through Bourdieu’s perspective, the relationships that the FTWs and NGOs developed with the community and patients, brought them social recognition that enhanced their *capital* over time. Bourdieu defines this as:

'The aggregate of the actual or potential resources which are linked to possessions of a durable network of more or less institutionalized relationships of mutual acquaintance and recognition – or in other words to membership of a group – which provides each of its members with the backing of the collectively-owned capital, a “credential” which entitles them to credit, in the various senses of the world. '[44:249].

In addition to the involvement of NGOs in the TB control programme, the Indian Medical Association (IMA) and private practitioners (PPs) were also considered to be important partners.

#### ‘We are not running a parallel programme…’

The IMA’s involvement was spoken of and supported by national programme managers and policy-makers. The IMA was actively involved in training allopathic practitioners. A representative of the medical association mentioned that ‘we are not running a parallel programme, but a support programme’. Similarly, PPs perceived themselves to be important healthcare providers, and viewed themselves in different roles within the PPM TB policy: *‘*to guide patients’; ‘to promote them’; ‘to save patients from every corner’; ‘to show them the right direction’, and so on.

…Very important, because the major chunk comes from private practitioners, people seldom go to big hospitals, corporate hospitals, and mostly they don’t prefer government institutions... [Interview, Dr. TA, Ayurvedic Practitioner]

The private practitioners considered themselves to be more accessible and approachable to patients when compared to the government institutions. Medical practice in general, all over the globe, holds a prime position in the professional hierarchy [55]. In his theory of professionalisation, Freidson [56] argued that ‘occupational groups, such as medicine, had previously engaged in a process of professionalisation to secure exclusive ownership of specific areas of knowledge and expertise’ [51]. Private medical practitioners in India continue to be respected based on their educational background and professional authority, and they hold ‘symbolic capital’ based on their prestigious position in society. In Bourdieu’s term, we can consider the educational background and professional authority of private practitioners as a form of capital. In addition, the profession is also associated with high financial gain which adds to the existing capital. The aggregate of these capitals that PPs hold together, have contributed to their social status and position within society.

The FTWs, PPs, and NGOs justified their own positions. Their ‘self-representation’^4^was not only a way to project their strengths but also a way to highlight weakness on the part of others.

### Unrecognized capital resources

Bourdieu (1984) cautions that the resources or capital one may possess in one field may be recognised or remain unrecognised in another field [42]. This theme demonstrates how having to engage with and work within the PPM-TB field, dislocated actors and therefore their social, cultural and symbolic capital retained within their individual fields was undervalued. Being undervalued, they felt demotivated and constantly struggled to find meanings in their association with the programme**.**

#### ‘We need freedom to prescribe …’

The experience of the PPs and their professional role in the medical field helps to shed more light on this dislocation of position. ‘Standard of care’ is the key factor that guides the medical profession [57], and is defined as ‘generally accepted principles for medical management’ [58]. Medical practitioners generally have the authority to make treatment decisions based on their individual professional judgement and expertise – and their ‘professional standing is rooted in its cultural authority, a form of legitimacy that enables physicians to make judgments of meaning and value and have these held to be true and valid’(Haplern [59] p.843). However, the educational background and professional status that PPs possessed within the medical field, was undervalued in the PPM-TB field.

For example, the entire idea of referring those suspected of having TB to government health facilities, and if diagnosed then to put them on standardised DOTS medicines, in a way challenged the traditional medical role of PPs as it gave them less freedom to prescribe or to decide on referrals. PPs asserted that such an approach in terms of their role in TB management not only decentralised the programme but also despecialised the skills of the PPs. A senior physician who has long-term experience of managing TB patients in his own clinics within the private regimen asserted:

...As a private doctor, I am not interested in 250/- Rs. What I need is the freedom to prescribe for my patients. [Interview, Dr VK, Allopathic Practitioner]

The ‘little flexibility’ in allowing PPs to prescribe TB treatment on their own, or using their own system of medicine as a supportive treatment (in the case of PPs trained in Ayurveda and Homoeopathy trained), can be seen as a process of undervaluing their educational capability as well as their professional skills. Our recent paper published elsewhere draws more attention to PPs perspectives about their involvement in the TB programme, highlighting the challenges of being undervalued by the programme [60]. Similar experiences, where PPs have felt undervalued in the TB programme, have been reported in other studies conducted in India and elsewhere [19, 61]. Although PPs got involved in the PPM-TB initiative, they were least interested or motivated by the financial incentives given by the programme.

#### ‘Our level is different; their level is different.’

Similarly, the position of FTWs was more complex in the PPM-TB field. FTWs are not primarily trained as marketing executives; rather, they are TB workers trained to work in TB units and DMCs, but also have to operate at the margins of the public-private interface. Having to engage with, and work within, the PPM TB field dislocates them. In the absence of cultural capital and knowledge of the policy, as well as a lack of skills in dealing with PPs, FTWs feared that the symbolic capital they possessed within the TU field was undervalued in the private sector field, giving them a sense of inferiority.

...Yes because as a Senior Treatment Supervisor, I cannot go directly to a private practitioner and tell him [to refer cases]…but if my Medical Officer comes with me and talks to the private practitioners, something may happen, that is there... our level is different; their level is different. [Interview, Senior Treatment Supervisor-2]

All FTWs, including the Senior Treatment Supervisors, often exhibited an inferiority complex when they entered the field of the PPs. This inferiority complex was greater in dealing with allopathic practitioners, who were considered to be busy, arrogant at times, and lacked interest.

[Excerpt from Field Diary, Thursday, 26 May 2011]

‘I accompanied the STS to the field for follow-up visits. As we entered the first MBBS doctor’s clinic, I saw the practitioner was sitting very professionally on his swivel chair, well dressed in a shirt and tie and a stethoscope hanging around his neck, and his arms resting on a clean table. The moment the STS greeted him, rather than offering a welcoming response, he took on a complaining tone. He very rudely informed the STS about how the DOTS patient had not been coming regularly to his clinic to take his alternate day doses, and as a result he wanted the STS to carry his box back to the TB unit and to deal with the patient accordingly thereafter. The poor STS had no voice nor authority to convince him, and had to take the box back with him.’

FTWs made distinctions between the allopathic practitioners and non-allopathic practitioners, mentioning that among the non-allopath, RMP doctors were well associated with the programme as compared to MBBS, MD, Chest Physicians. In an FGD, all FTWs jointly voiced that, ‘In this programme 80 % help is from RMPs only…but…from specialists 0 % support…’.

#### ‘Government agreements are more of an entanglement…’

NGOs were working in close proximity with the community and patients and regarded themselves as the crucial link in the entire programme. In Bourdieu’s term these relations that NGOs developed with the community and patients brought them social recognition that enhanced their Social Capital over the time. However, lack of acknowledgment and least support by the district authorities undermined NGOs social capital that they carried with them to the programme.

NGO Partners felt that the system failed to recognise their contribution, to the extent that the district programme managers failed to acknowledge their efforts and hard work. In the absence of a proper referral recording system at the DMCs, partners felt de-motivated. They questioned the value of the partnerships and whether the government was truly committed to it or not; however, whenever an NGO contributed at the field level, it was always taken as the work of the government.

…They [programme managers] are not at all open in sharing their opinion about the partners' contribution [to the programme]. Some programme managers acknowledge the contribution, but some still say that, “Ok, NGO-C has done some activities, but mostly they [government] are contributing…” [Interview, Project Coordinator, NGO-C]

The experiences of the partners concerning the participation of the DTOs and their unresponsive behaviour, led them to feel that their relationships with the programme was undervalued and denigrated. During 14 months of fieldwork, it was observed that, four District TB Officers (DTOs) had been transferred. Partners identified a change in the DTO’s leadership as a process where responsibility was transferred to Collectors and non-state partners, limiting the DTO’s power and authority to make decisions. Partners asserted that the Collector, being the chairperson of the District TB control society, had the sole signatory power over signing of the MoUs. Partners expected renewal to happen automatically based on their performance and along the guidelines, however, considering the busy schedule of Collector, the renewal of MoUs got delayed upto several months.

...our role is to mobilize communities, NGOs, CBOs, to sensitize them and they have applied for the schemes, but it is lying at the district control society because the initiative has not been taken by the DTCO or if the DTCO takes the initiative then it is blocked at the Collector’s level… [Interview, Director, NGO-A]

NGO-A holds a long-term relationship with the programme in terms of supporting them by running five DMCs across the district. However, it was observed that the Project Associate at the field office had to make several visits to the district TB Centre (DTC) to get the MoUs signed.

The signing of MoUs is associated with the release of funds. The delay caused in getting the MoUs signed prolonged the release of funds. NGO-A mentioned that for them, being a big organisation, in times of delays they continued to manage their activities with money they received from other independent projects within the organisation. However, they felt concerned about several other grassroots organisations whose survival depended on the programme funds only.

Partners felt that these hurdles not only delayed the process, but also created an uncertainty about whether the MoU would be renewed or not, or whether finances would be released or not. That is the most pressing question for the NGOs, and its effects tend to demoralize the NGOs, giving them a feeling that ‘getting into an agreement with the government is more of an entanglement’.

We can say that the diverse groups of actors entrusted with carrying out the implementation of the TB partnership were operating within the capital resources available in their respective fields. However, their contributions towards programme implementation tended to be poorly recognised, placing them in a position where they felt inferior to other partners.

### PPM-TB field: ‘site of struggle’

In Bourdieu’s work, a field is a ‘site of struggle’ in which various social actors compete over, contest and construct and influence power, to preserve or overturn the existing distribution of capital. As a response to this misrecognition in the PPM TB field, and in order to maintain their positions, frontline actors used various mechanisms and strategies at the individual and organisational levels.

One of the critical properties of the field, as mentioned by Wacquant [39:8] is also ‘its degree of autonomy, i.e., the capacity it has gained, in the course of its development, to insulate itself from external influences and to uphold its own criteria of evaluation.’ The medical profession in India is a diverse group of practitioners, each practicing different systems of medicine. The market forces govern the private sector and breed competition so that the practitioners are forced to adapt and to create space for themselves [62].

The IMA can be considered as an individual field within the private medical sector. It enjoys its autonomy based on cultural and symbolic capital. The professional identity and authority that the IMA retains in its mindset can be witnessed in their actions at association level, as well as in the actions of individual practitioners.

### IMA professionalism

At the national level, it is the IMA that takes responsibility for involving the allopathic practitioners. The IMA has played a powerful role in maintaining the status quo of allopathic medicine (western medicine), to such an extent that they have opposed those outside the purview of allopathic medicine from practicing allopathy. In recent years, the IMA has strongly lobbied against the legalisation of unqualified medical practitioners and their utilisation in any health programme [63–65] and have even dismissed them as ‘qualified quacks’ [59].

Involvement of non-allopathic practitioners still does not constitute a strong focus at the policy level, and thus they remain excluded from the policy stream. A policy-maker, commenting on the involvement of non-allopathic practitioners, acknowledged that they are the major source of referral of TB patients and their contribution to the programme is substantial. However, the policy-maker had reservations about legally incorporating the involvement of non-allopathic practitioners into the written policy documents, due to pressures from the IMA.

...When we were writing up private practitioners schemes, we refrained from putting that [involvement of informal providers], because the moment it enters into the government document, the IMA will come down strongly against us, saying that we are legalizing quackery. So it will not be in any written documents, but the fact is that we are very well aware of it and we recognize their work in the field. A huge chunk of TB patients goes to them only, they are the first point of contact, so we always say and we always advise the programme officers, that they have to be included, they should be the DOTS provider, or the referral points. But you will not find it in any of the schemes as such, because then it is against the ethics... [Interview, National level PPM-TB Consultant -2]

NGO-B, based on their field experience of training the non-allopathic practitioner’s, elaborated on this gap in policy, where non-allopathic practitioners were never considered as legitimate potential partners.

...We identify RMPs, whether the government recognizes or not. You know the medical call, I mean the institute, they don’t count them as real partners in the RNTCP... [Interview, Programme Officer, NGO-B]

IMA’s ability to maintain their professional authority is based on their educational and professional profile. It not only hinders the TB programme’s ability to reach them, but also obstructs the programme’s relationship to those outside the allopathic system. Their professional authority has hindered the potential of creating a partnership policy that encompasses all healthcare providers.

### Private practitioners authority

In terms of the PPM-TB policy, allopathic practitioners fail to participate fully because of the programme’s inflexibility in prescribing private treatment. However, they continue to enjoy their autonomy in their capacity to self-manage TB cases by referring only those cases which they feel need to be referred.

As per the RNTCP guideline, the DOTS provider is expected to ensure follow-up sputum collection, late patient retrieval, maintain RNTCP records for the patients, and permit on-site monitoring by RNTCP supervisory staff. FTWs, however, had observed that the PPs considered the updating of cards to be an additional task and often showed unwillingness to keep the DOTS boxes. However, in the interest of the patient’s convenience, FTWs had to negotiate with the PPs and take up the responsibility of updating the cards, leaving the responsibility of dispensing the medicines with the PPs. This was observed on one occasion when the researcher accompanied the field staff for a follow up with a PP DOTS provider at his clinic.

[Excerpt from Field Diary, Friday, 4 February 2011]

'Dr KMC is a senior homeopathic doctor. When we entered the clinic he was setting the medicine on the shelf. FTW greeted him and spoke a few words and came out of his cabin. In the waiting hall there was a metal cupboard [usually a book rack] in which all medicines were kept. Over this cupboard two DOTS boxes were placed. FTW stretched out his hand, pulled the first box and placed it on the bench. He opened the box; the duplicate treatment card was folded inside it, and was covered with dust on one side. FTW wiped off the dust with his hands and unfolded the card to see the tick marks. There were no tick marks after Dec. 2010. He counted the strips and looking at me commented, "patients drugs *nahi kha raha*" ['patient is not taking the drugs']. He then dialled the Lab Technician’s number at the DMC and asked him to give him the details (dates and results) of sputum follow-up test of patient number 462. The technician gave the results and FTW updated the treatment card. He closed the box and placed it back on the cupboard, and then repeated the same procedure for the next box.'

Although PPs who were referring patients to the DMCs/ DOTS programme, they enjoyed their autonomy in terms of which patient were to be sent and which would not be sent. They supported DOTS in principle, but in practice made no efforts to counsel and encourage patients to go to the DOTS centres. Although PPs mentioned their role in counseling the patient and giving them a choice to select their treatment provider based on affordability, patient decisions were mostly influenced by PPs own individual judgements.

### FTW discretion in approaching PPs

FTWs were often working in resource-limited settings and, at the same time, were pressured by programme policies. A lack of human and financial resources not only hampered routine ‘fieldwork’, but also demotivated them from taking on new activities [66].

As a routine practice, and based on their experiences with PPs in general, FTWs occasionally only visited PPs who had DOTS boxes in their clinics and often excluded those who were not keeping DOTS boxes. Furthermore, there were no active attempts by programme staff to go and sensitise PPs who had never been DOTS providers or referred any cases, leaving a large number of PPs outside the purview of the programme. One day while in the field, it was observed that besides a DOTS provider’s residence, there were two other clinics run by MBBS doctors. The researcher looked at the fieldworker and asked him, ‘do they have any of our DOTS boxes?’ He replied, ‘I never went there.’ One reason for this kind of approach may be attributed to not having appropriate training relevant to the PPM-TB policy.

The involvement of PPs was seen primarily from the perspective of simply meeting targets, rather than building a long-term relationship to enhance capacities. One afternoon the first author was returning with a member of the field staff from a field visit. During the conversation, he posed an informal question: ‘So do you go to see these PPs regularly?’ The field staff member replied, ‘If fewer numbers of patients come then we go around in the field to visit private doctors in this area ‘. This understanding of visiting PPs just to achieve targets was infused into their day to day practices.

…To involve means increasing the cases...what doctors [Higher Officers] tell us is that at each centre there has to be minimum of 10-15 sputum positive cases… from ‘Out Patient’ we only get 2-3 cases. So to get more cases we need to approach the PPs... [Interview, TB Health Visitor-2]

It was surprising to observe in the above quote that this mind-set that the PPs need to be approached only when the programme needs additional cases, originated from the top level, showing that there is a poor understating of what this partnership entails. Throughout the fieldwork, the researchers did not find any new PP being added to the list of DOTS providers, neither did they witness any FTW approaching a new PP to sensitise him/her about the TB facilities at the DMCs.

In principle, FTWs approved of the PPM-TB policy, however, in practice they showed a negative attitude towards PPs and NGOs, and limited the amount of field visits they made. Other studies show similar behaviour amongst frontline staff [67, 68]. Field observations and informal interactions with actors have shown that the integration of the PPM-TB policy into routine TB work focused little on building effective relationships.

### The autonomy of NGOs

As discussed in the earlier sections, NGOs capital, on the one hand, was undervalued by the public sector in the PPM-TB field, while the public sector and the funders still expected them to perform well. NGOs often struggled to strike a balance between fulfilling the expectations of the programme and meeting the performance targets of their funders. As coping strategies, firstly, they developed their own mechanisms to achieve the targets, and secondly, to maintain their identity they kept a distance from other partner NGOs (least collaborating with them). To give an example, a senior public sector Chest Physician (CP) who also holds an established private practice, regularly referred his TB suspects to the NGO run DMC for sputum testing. Once tested and based on his judgement, he asked the patient to either continue the treatment at the DMC or to start the TB treatment at his clinic. On average he referred 25% of diagnosed cases to the DMC for initiation of DOTS. Being associated with the public sector, he understood that the core value of sputum examination is to correctly diagnose TB. However, he was not in favour of the RNTCP alternate day regimes.

In accordance with the RNTCP guidelines, any client that gets tested has to be first registered in the Outpatient register, followed by the lab register. If the client is sputum positive, s/he has to be put on DOTS, which is the responsibility of the frontline staff at the DMC. If the Chest Physician refers a case, and the case is diagnosed as sputum positive, how does the person begin treatment, and how does the Lab Technician manage the register? A Lab Technician mentioned that whenever a case was referred by the Chest Physician, he did the investigations first, without registering the patient details in the lab register. Once TB diagnosis was confirmed then he consulted the Chest Physician and if he decided that the patient could be retained by the DMC, only then did the Lab technician registered the client. In contrast, if the suspect was diagnosed to be without TB, then the Lab technician registered the client without consulting the Chest Physician. This also meant that patients who continued to be treated by the Chest Physician were confirmed to have TB by the DMC centre first, yet they were not documented in the lab registers.

The Laboratory technician was asked how this action benefited the DMC. He replied that the Chest Physician refers a substantial number of cases for DOTS. If they were to ignore testing his private clients, then the NGO might not get any referrals from him in the future. The NGO-A DMC needed cases to achieve quarterly targets and the Private Practitioners needed business. This situation revealed how the situation was managed mutually and informally by going beyond the policy norms. The Private Practitioners brought their professional authority to bear, making the NGO-DMC dependent on them for their case load.

The informal referral model between the PPs and NGO-DMC provides a crucial learning scenario. From a top-down (structural) perspective, it can be viewed as a violation of the PPM-TB policy guidelines, however, from a bottom-up perspective (agency) it was successful, as it maintained strong relationships between the PPs and the programme. Both the NGOs and the PPs mutually benefitted from it and ultimately delivered the timely service required by their clients. This story demonstrates Susan Barrett and Colin Fudge’s characterisation of the relationship between ‘policy and action’ as a negotiation process [26].

According to Bourdieu’s theory, the Frontline workers were rewriting the rules of the game in order to safeguard their ‘position’ in the field and to augment the ‘position’ of the TB Unit in comparison to the other TB units. They were breaking the rules by limiting field visits, developing informal links with PPs, and least coordinating with NGOs, which itself had become the ultimate metric in the game operating out at the TB unit.

## Conclusions

The nature of this study was exploratory. The main focus was not on assessing policy outcomes in terms of successes or failures, but on understanding the individual voices and challenges different actors face, and the effect on partner relationships. Drawing on an anthropological approach it explored the social relationships and interactions among the health workforce, the values and meanings ascribed to those relationships, and to the power dynamics within the health system at the local level.

The strength of the study lies in the insights it provides through observations and informal interactions made in the field on a day to day basis as well as its multi-actor perspective. Unlike other policy implementation studies where the focus of the work has been on single actors - e.g., frontline workers [68], programme managers [69], and health care providers [70] – this study adopted a multi-actor perspective across different sectors, seeking to provide a more holistic understanding of the implementation process of the PPM-TB policy.

The study found that programme managers, frontline TB workers, NGOs, and private practitioners all had a crucial role to play in TB partnerships. They were widely regarded as valued contributors with distinct social skills and capabilities within their organizations and professions. However, their potential contributions towards programme implementation tended to be unrecognized at the bottom level of the policy implementation chain. These actors constantly struggled for recognition and used different mechanisms to position themselves alongside other actors within the programme that further complicated the relationships between different actors. To summarize the analysis, PPM-TB can be seen as ‘the field’ that in itself represents an intersection between the relatively discrete fields of the public, private and NGO sectors. Each of these discrete fields had individual actors operating within them, who also came to play an important role in the shared field of PPM-TB. Due to the varying degrees of ‘capital resources’ available in their particular field – as well as the shared field – they were constantly positioning themselves in relation to other actors. Although the different actors were widely regarded as valued contributors who possessed distinct social skills and capabilities within their organizations and professions, their potential contributions towards programme implementation remained poorly recognized, making them feel they were being poorly treated. As a result, all actors constantly struggled to find meanings in their involvement with TB programme and used their different coping strategies to retain their positions in the shared field of PPM-TB: FTWs approached PPs selectively; PPs avoided referring patients; the IMA forced measures at the policy level to discredit non-allopaths; and NGOs developed their own referral models.

The application of Bourdieu’s theoretical insights in this analysis has specifically enabled us to provide a contextual understanding of the complex process of policy implementation at the local level. These insights have helped us go beyond the field description and look into the social realties hidden in the implementation process of public-private partnerships for TB control. They have specifically helped reveal how the social practices of actors became routinely embedded and integrated into their social contexts, and the way they searched for meanings to characterize their relationships.

The study contributes to our understanding of implementation of PPP for TB control and builds knowledge to help policy makers and programme managers strengthen and effectively implement strategies to enable stronger governance of these partnerships. The actors entrusted with carrying out the implementation of a policy or programme, operate within the capital resources available to them in their particular professional field. A policy or programme can only succeed if the positions of those implementing it are recognized by showing respect to them, and by indicating that the programme needs their active participation. Such recognition needs to go hand in hand by truly involving them, which could include incentives (both economic and non-economic) for their efforts. This might increase their enthusiasm and make them feel ‘responsibility’ towards the policy/programme they are involved in implementing. If this enthusiasm is supported by a strong shared vision, through imparting adequate information about the policy intentions, it will bring meaning to their relationships with other actors, thereby contributing to a strengthened system.

This above paragraph identified some key relational elements in the implementation of PPP. These relational elements are interlinked, exert influence on each other, and are complex in nature. Further studies on PPPs should take into account these elements that would help build a framework for evaluation and implementation of PPPs.

Ultimately, the paper argues that in ethnographic ‘implementation’ studies with multi-actor perspectives, a focus on individual and organizational positions in relation to others helps to provide a better understanding of the complex relationships found in partnerships policies (in low and middle-income countries). This more comprehensive understanding of these processes is a prerequisite for strengthening local health systems through more effective partnership strategies by bridging the gap between field-level practices and central policy intentions.

## Abbreviations

DMC: Designated Microscopy Centre
DOTS: Directly Observed Treatment Short-course
DTO: District Tuberculosis Officer
FGD: Focus Group Discussion
FTW: Frontline TB Worker
IMA: Indian Medical Association
NGO: Non-Government Organization
PP: Private Practitioner
PPM: Public–Private Mix
PPP: Public Private Partnership
RNTCP: Revised National Tuberculosis Control Programme
RMP: Registered Medical Practitioner
STS: Senior Treatment Supervisor
TB: Tuberculosis
TU: Tuberculosis Unit

## Footnotes

1 WHO defines public-private mix (PPM) for TB control as ‘strategies that link all healthcare entities within the private and public sectors (including health providers in other governmental ministries) to national tuberculosis programmes for expansion of DOTS activities’ [4:1].

2 The Designated Microscopy Centres (DMCs) are the crucial places where the TB suspect or a TB patient first comes into contact with the TB programme. The DMCs could also be seen as a “gateway” or an entry point for the TB suspect or a TB patient to access the TB diagnostic and treatment services offered by the RNTCP. Any TB suspect or a TB patient has to go via this route to be officially enrolled in the TB programme.

3 Fifteen Primary Health Institutions (PHIs); three Civil Dispensaries (CDs); five Urban Health Posts (UHPs); one Area Hospital (AH); and one Employees’ State Insurance (ESI) Hospital.

4 Self-Representation: As per the Gale Dictionary of Psychoanalysis, self - representation is ‘the image the subject has of him or herself based on his her own interpretation. It is one of the factors of the ego and its representation as termed “an individual, differentiated, real, and permanent entity” (Racamier) particularized by a distinctive history and modes of feeling, thinking, and doing.’ (Gale T. International Dictionary of Psychoanalysis, 1st Edition. Macmillan Reference USA; 2005.)
